# Loot

**Published:** 1869-12

**Authors:** 


### Loot.

### *Malarial Hæmaturia.*

OUR readers are aware that since 1866 there has been prevailing, through different sections of the Southern States, a peculiar form of malarial disease, to which this designation has been affixed. It has been variously termed: "Hæmorrhagic malarial fever, black jaundice, cachæmia or malarial cachæmia, cachæmia hæmorrhagica, malignant congestive fever, icterode pernicious fever, purpuræmia, yellow remittent, yellow disease, canebrake yellow fever, new disease, intermittent icteroid fever, malarial hæmaturia, bilious hæmaturic fever," etc.

From an intelligent editorial in the *Southern Argus* (Selma, Ala.,) we clip the following:

"It naturally occurs to us to ask why we should have a new and dangerous disease amongst us since the war, in addition to all our other troubles? Perhaps a good reason can be found. Our surrender, in 1865, has been the 'direful source of woes unnumbered"—political, social and pathological. Among the pathological woes is this disease; and it is consequent upon the others. The exclusive cause of this disease, as it is of fever and ague, chills, congestive chills, bilious fever, etc., is *malaria*. Of course we can not tell what malaria is; but we can tell what are found to be the conditions necessary for its generation, to-wit: heat, moisture (stagnant), decaying vegetable matter, and a long-continued still or motionless atmosphere, or absence of strong air currents and winds. These conditions have been more abundant in the South since the war than previously. The deranged labor system has left large tracts of land, previously fine plantations, to lie idle, the ditches on them to become filled, and the water drains generally clogged up; ponds have formed where once were fields; in other words, an efficient policing and hygiene of plantations and towns has been neglected. In this way our climate has been caused to resemble that of the warm infra-tropical countries; and with this state have come the diseases hitherto confined to those regions. All the diseases of our section are influenced by this atmospheric constitution. We have more of the graver forms of malarious diseases than of yore. It may be that the depression of spirits of our people has had some agency in this, in addition to the bad atmospheric constitution. They have co-operated. Melancholy and despondency always invite low forms of disease, and make the human body much more susceptible to attacks from morbific poisons. Thus comes about this fatal disease. The bad *morale* of our people cripples their vital energy, and makes them an easy prey: our neglected plantations and insufficient sanitary regulations and hygienic policing have made malaria more abundant and potent; so, these two causes combining, we have all the malarious fevers we have heretofore been used to in a graver and less manageable form, and, in addition thereto, a new and grave disease, of malarial origin, invited up from the tropics. This disease is not the yellow fever. There are two forms of yellow fever recognized in Aitken's work on the Practice of Medicine, viz.: specific yellow fever (the worst kind), and malarial yellow fever. These come as epidemics. The disease under consideration is neither one of these. The reader can select any of the names for it proposed above that suits his taste; though it would be well not to call it yellow fever, for fear of confounding it with the genuine disease of that name.

"We do not venture to suggest a treatment for this disease; for in this the ablest members of the medical profession fail to agree among themselves; but we believe it is agreed that prevention is easier than cure, and that this disease can be prevented. The way to exterminate the disease is to enforce throughout the country the most rigid sanitary regulations, in the matter of ditching and draining especially. The way to prevent this disease in each individual case, is to cure the chills, to prevent the deterioration of the blood by long exposure to malarial influences. Chronic chills are the finger-boards to malarial hæmaturia; and this fact is at once the key to its cause, and the guide to a rational treatment. Almost invariably the victims of this disease have been long affected with malarial poison, if they have not had regular paroxysms of intermittent fever."

***Embolism of the Heart and Pulmonary Arteries.***

PLAYFAIR thinks that the term *embolism* should never be applied to cases of fibrinous obstruction of the heart and pulmonary arteries, uuless two conditions are met with on *post-mortem* examination. One is the existence of a clot in some part of the peripheral venous system, from which an embolus can be derived; the other is the existence in the pulmonary clots of distinct and separate portions of peculiar fibrine, which has traveled from the peripheral thrombus, to which it corresponds in structure, and around which more recent fibrin is generally deposited. If no clots exist in any of the veins, the case clearly cannot be one of embolism, but of thrombosis.

Embolism of the pulmonary artery is a frequent cause of sudden death after delivery, and Playfair reports a case in which the traveled embolus was not only found, but the exact site from which it was detached also determined. A young woman, in the sixth month of her third pregnancy, underwent an operation for fissure of the rectum; abortion took place on the eighth day, preceded by a pulse of 160, temperature of 103, and respirations 40. Then she had extreme dyspnœa, face excessively pale, and expressive of extreme anxiety, lips white, uterus well contracted, no pulse at wrist or posterior tibial artery, the sounds of the heart almost *nil;* in fact, she had all the symptoms of obstruction of the right side of the heart characteristically developed. She called incessantly for air, and died as if suffocated.

The right side of the heart was extremely distended, as were, also, the large veins of the neck and the two cavæ. At the bifurcation of the pulmonary artery, plugs of firm fibrine were found extending into the primary divisions of the pulmonary artery. In the centre of one of these fibrinous plugs was found another piece of fibrine, of the shape of an almond, very rough on one side, nearly an inch long, surrounded by, yet separate from, the remainder of the fibrine, which was of a less firm consistence.

At the junction of the internal and external iliac veins of the right side, there was a clot of fibrine about two and a half inches long, and about its centre was a roughened portion which fitted closely to the corresponding rough surface of the pulmonary clot.

It is very clear what had occurred. At some period of the eight days which elapsed between the operation and death, a coagulum had formed in the iliac vein. The portion found in the centre of the pulmonary coagulum had become detached, and was carried through the venous circulation until it was arrested in the pulmonary artery; then fresh fibrine was deposited around it until the pulmonary arteries became more and more obstructed, and death ensued.

In twenty-five other cases, twelve only were due to true embolism, and death generally took place after the nineteenth day; the remaining fifteen were cases of thrombosis, in which the fatal result occurred at a much more early period.—*Med. Gazette.*

### *Aqua Chlorini in Trichinitis.*

DURING the late epidemic of trichinitis in Hildesheim, Germany, one of the physicians, taking the symptoms of his three first patients for cholerine, prescribed concentrated *Aq. chlorinii*, and noticed rapid subsidence of the colicky pains. He continued this remedy even after observing the mistake, and succeeded with it fully in fourteen cases, five of which were of the highest grade, by giving at first, every third hour, two tea-spoonfuls, and when the catarrh of the stomach had gradually subsided, one tea-spoonful, a draught of water to be taken five or ten minutes after the dose.—*Med. Bulletin.*

### *Deodorized Carbolic Acid.*

CARBOLIC ACID may be deodorized by mixing it in a crystalized form with twice its weight of gum camphor, and adding whiting to the compound. In this form it is said to be valuable, both as a disinfectant and as a protection to furs against moths.—*Med. Bulletin.*

### *Treatment of Delirium Tremens.*

DR. FLEMING, of the Queen's Hospital, Birmingham (*British Medical Journal*), treats delirium tremens as follows: The patient is placed in bed. For a diet, milk and a strong beef-tea, alternately, every four hours, and gradually, as the appetite improves, chicken, mutton, etc., until the stomach can accept ordinary food. Alcoholic stimulants in every form are stopped at once and entirely. When the patient is faint the following is prescribed:

℞ Ætheris chlorici (Duncan & Flockhart), spiritus ammoniæ

aromatici, sing., - - - - - - - ʒ iij;

Tincturæ lavandulæ, comp., - - - - - ʒ iv;

Spiritus vini gallici, - - - - - - - ʒ x;

Two drachms for a dose, in a wine-glass of water, every two or four hours.

The further addition of alcoholic poison having been stopped, the objects of treatment are: First, To eliminate the poison already in the blood. Second, To control its effects. Third, To favor convalescence. To promote elimination by the skin and kidneys, the following mixture is given:

℞ Spiritus ætheris nitrosi, liquoris ammoniæ acetatis, sing., ʒ v;

Soda phosphatis, sodæ et potassa tartratis, sing., - ʒ v;

Aqua ad., - - - - - - - - - ℥ xx; M.

Two ounces every four hours, two hours before meals.

A colocynth and hyoscyamus pill, with from ½ grain to a grain of the acetic extract of colchicum is given, should an active purge be indicated. To control the effects of the poison two drachms of the following are to be taken every four hours, one hour before food, in a large wine-glassful of water: Ten drachms of dilute phosphoric acid, with twenty drachms of tincture of hops. To induce sleep, the subjoined draught is ordered:

℞ Ticturæ cannabis, - - - - - M. xxx. ad lx.;
Liqoris morphiæ acetatis, - - - - M. xv. ad lx.;
Spiritus ætheris nitrici, - - - - - - - ʒ i;
Aquæ pimentii, - - - - - - . - - ad ℥ ij; M.

This medication is pursued until convalescence is established, when the tonic regimen is strictly enforced, incuding pure air, good food, cold bathing, with zinc and iron as blood tonics.—*Med. Record.*

### *Detection of Blood Stains.*

Dr. Richardson, in the *American Journal of Medical Science*, speaking of this subject, says:

Through the courtesy of Dr. Linderman, Director, and Mr. J. R. Eckfelt, Chief Assayer of the United States Mint, I was enabled to estimate the delicacy of the microscopic test for blood, as follows: Upon a square of waxed paper determined by Mr. Eckfelt, on the accurate balance used for the National Assays, to weigh exactly 48 milligrammes, I made twenty dots of fresh blood from my finger, which, when dry, added .4 of a milligramme to the original weight, and consequently were each on an average equivalent to about .02 of a milligramme, or 1-3200 of a troy grain nearly. The fourth part of one of these spots, weighing, of course, in round numbers 1-12000 of a grain, was detached with the point of a cataract needle, and, when moistened under the 1-25 showed many hundred well-defined, red-blood corpuscles; ten circular ones among them, measured with the micrometer, averaged 1-3494 of an inch in diameter, and could, therefore, by this criterion of superior size alone, be diagnosticated from the corpuscles of an ox, sheep, or pig, with the same feeling of certainty with which any surgeon could testify that a perforation of the skull only half an inch across could not possibly have been made by any bullet measuring an inch in diameter.

The *Boston Journal of Chemistry*, on the same subject, says: Prof. Bloxam has found that a mixture of tincture of guaiacum and ozonized ether (that is, a solution of peroxide of hydrogen in ether) instantly produces, with blood or blood stains, a beautiful blue tint. Prof. B., in the case of a stain twenty years old, extracted a single linen fibre with an almost inappreciable amount of the stain upon it; and, on subjecting it to this test, the blue color was readily distinguished through the microscope.

The spectroscope may also be employed as an extremely delicate test for blood-stains. Mr. Sorby has shown that the one-thousandth part of a grain of the red coloring matter of the blood may be thus detected with the greatest certainty.

### *Recipe for Burns.*

Make a saturated solution of alum (four oz. in a quart of hot water). Dip a cotton cloth in this solution and apply immediately on the burn. As soon as it becomes hot or dry, replace it by another, and continue doing so as often as the cloth dries, which at first will be every few minutes. The pain will immediately cease; and, after twenty-four hours of this treatment, the burn will be healed, especially if commenced before blisters are formed. The astringent and drying qualities of the alum will entirely prevent their formation. The deepest burns, such as those caused by boiling water, drops of melted metal, phosphorus, gunpowder, fulminating powder, etc., all have been cured by this specific.—*Ibid.*

### *The Case of Dr. Schoeppe, of Carlisle, Pennsylvania.*

STATEMENT of a Committee of the Medical Society of the County of New York, in relation to a "Hypothetical Case," presented by Dr. Schoeppe, and based upon the testimony of the witnesses during his trial.

The papers forwarded by Dr. Schoeppe were presented at the stated meeting, September 6, 1869, the earliest moment practicable after their arrival, and referred to a committee of three, with power. The following is the result of their deliberations:

ABSTRACT OF THE EVIDENCE. — The only evidence in relation to the matter examined by the committee, is found in four octavo pages of printed matter, entitled, "Hypothetical Case based upon Testimony of the Witnesses," and "Chemical Analysis based upon the Testimony of the Witnesses." It is therein shown that a woman, sixty-five years of age, a frequent sufferer from vertigo, complained of illness thirty-three hours before her death. She was probably nauseated, since an active emetic was taken, which produced its legitimate result about thirty-one hours before death. Eight hours after this, or twenty-three hours before death, she became semi-comatose; coma was pronounced in about three hours more; and twelve hours before death paralysis of the right side of the face and tongue had supervened. Paralysis of the entire right side of the body is rendered highly probable by the position in bed, and the warmth and moisture of the right side, as contrasted with the coldness and absence of perspiration on the left. Both pupils were contracted. The patient remained in this condition until her death. The positive symptoms were these: Old age, vertigo, "indisposition," vomiting, semi-coma merging into coma, paralysis of right side of face, difference in temperature of two sides of body, and contraction of pupils.

That feature, in this train of symptoms, which must at once attract attention, is the paralysis. Exclude this from the evidence, and the remaining signs would indicate either uræmic or narcotic poisoning. But with paralysis of *one side* of the body present, even more strongly than if it existed on both sides, poisoning by any medicinal agent is at once imperatively excluded. No drug, or combination of drugs, is capable of destroying the nervous power of a portion of the body situated at one side of the median line, while this power is retained by the corresponding part on the other side.

And yet this is precisely the condition, which lay witnesses have shown, obtained in the case above quoted. The right side of the face lost its nervous stimulus, and became incapable of resisting the normal power of the opposite muscles. These, therefore, dragged the mouth and nose toward the left. The tongue was in a similar condition. It is not necessary to refer to the remainder of the body, for though it is probable that hemiplegia existed, it would not strengthen our position were the proof complete.

That the symptoms were due to blood poisoning consequent upon kidney disease, is possible, but not probable, since death from uræmia is commonly ushered in by convulsions. We have a much more likely cause in either softening or sanguineous apoplexy in the brain. The former is a recognized cause of all the symptoms in this case, and upon inspection the diseased organ may yield no other evidence of the change than a reduction of the normal firmness of the parts.

This brings us naturally to the autopsy, which was conducted in a deplorably negligent and unscientific manner. The examination of the brain revealed an absence of effused blood in the substance of the organ, and also from its superficial parts, unless the "large amount of blood in the cranium, the origin of which the examining physician could not tell," were clotted. This, however, is unlikely, both from the fluidity of the blood in other parts, and the fact that apoplexy was not therefrom suspected. A portion of the brain near the fourth ventricle was found to be softened, but twelve days after death, even in winter, a slight degree, at least, of such change might be expected. We therefore attach very little importance to this discovery. The arteries of this organ should have been carefully traced, since a coagulum forming suddenly in one or more of them would be likely to produce very sudden death with vertigo, nausea, paralysis and coma. The softened part should have been submitted to microscopical investigation. The heart should have been scrutinized with utmost care as to the condition of its valves and its size; and the arteries with regard to their coats; especially in the kidneys of so aged a person should have been sought some explanation of the phenomena. All of these points were omitted, and we have the single item of positive intelligence, of questionable value, that softening existed.

To return — cerebral apoplexy is probably excluded, and we are brought to the conclusion that white softening, or partial anæmia of the brain, was the cause of death. With regard to the *post-mortem* appearances in this affection, we may quote from one of the highest authorities in matters of this nature, Professor Niemeyer, who states that "Partial anæmia of the brain can not by any means always be definitely made out in the dead body. * * * On account of the difficulty of deciding whether a portion of brain has been anæmic during life, we should make it a rule to seek most carefully for any obstruction of the cerebral arteries, particularly of the arteria fossæ Sylvii, in any cases where the patient has died from a chronic brain disease, or an acute one beginning with a sudden occurrence of hemiplegia, unless the autopsy gives some other satisfactory explanation of the symptoms. Before attention was called to the occurrence of this anomaly, autopsy, in cases of severe brain disease with hemiplegia, often furnished nothing satisfactory."

The same author quotes Bamberger with deference, as expressing the opinion that it is well nigh impossible, in cases of sudden death preceded by hemiplegia and coma, to predict whether sanguineous apoplexy or partial anæmia would be discovered upon examining the brain.

As may be inferred from the first quotation, white softening of the brain is due to the more or less rapid plugging of cerebral arteries by clots of blood or fibrin, which thus cut off the vascular supply, and produce death (necrosis) of the brain tissue to an extent commensurate with the distribution of the artery or arteries involved. This may be caused by a diseased condition of the walls of the vessels of the brain, or by a plug coming from the heart or lungs.

The prominent symptoms which announce this condition are vertigo, which may have existed for many months, nausea, and finally paralysis, coma and death.

It is therefore our opinion that the patient died from natural causes, judging exclusively from the "Hypothetical Case" presented to us.

Wm. B. Lewis, M.D.,

Emil Neoggerath, M.D.,

A. Flint, Jr., M.D.

—*Medical Record.*

Dr. Schoeppe has been sentenced to death, but we believe Gov. Geary has temporarily suspended the execution. So far as the published evidence goes, we most fully believe that his hanging would be nothing more nor less than judicial murder.

***Lactic Acid in Croup.***

℞. Acid lactici gtt. xv ad gtt. xx; aq. ℥ ss M., may be used to advantage in croup. This quantity to be used every half hour with an inhaling apparatus, diminishing the acid to gtt. x, then to gtt. v, and inhaling it but every half hour or two hours, when the dyspnœa decreases, which is said to disappear entirely in seven, ten, or twelve hours. Protect the eyes and face from the vapor. Dr. Weber prescribes, besides this local treatment, the following internally: ℞. Sodæ bicarb., ʒ ij; aq. ℥ iv. M. Sig. A table spoonful every half hour, or every hour, till the dyspnœa disappears.—*Med. Gazette. Med. Bulletin.*

***Milk.***

Dr. Cameron, a Dublin chemist, has been experimenting on the milk of the sow, and has shown that it contains eighteen per cent. of nutritious matter, while human milk contains but eleven per cent. The relative nutritive values of different kinds of milk may be thus arranged in an increasing order: Human milk, cow's, goat's, ewe's, mare's, ass's, and sow's. This accounts for the rapid growth of young pigs compared with other animals.

